# Clinicopathological hallmarks and biomarkers of colorectal neuroendocrine neoplasms

**DOI:** 10.1371/journal.pone.0188876

**Published:** 2017-12-12

**Authors:** Alexander Koenig, Sebastian Krug, Daniela Mueller, Peter J. Barth, Ute Koenig, Michael Scharf, Volker Ellenrieder, Patrick Michl, Roland Moll, Kia Homayunfar, Peter Herbert Kann, Philipp Stroebel, Thomas M. Gress, Anja Rinke

**Affiliations:** 1 Department of Gastroenterology and Endocrinology, Philipps-University of Marburg, Marburg, Germany; 2 Department of Gastroenterology and Gastrointestinal Oncology, University Medical Center Goettingen, Goettingen, Germany; 3 Department of Internal Medicine I, University Halle, Halle, Germany; 4 Gerhard-Domagk-Institute of Pathology, University of Muenster, Muenster, Germany; 5 Department of Pathology, Philipps-University of Marburg, Marburg, Germany; 6 Department of General-, Visceral- and Pediatric Surgery University Medical Center Goettingen, Goettingen, Germany; 7 Department of Pathology, University Medical Center Goettingen, Goettingen, Germany; University of South Alabama Mitchell Cancer Institute, UNITED STATES

## Abstract

Chromogranin A (CgA) is a well-established marker for diagnosis and follow up of patients with gastroenteropancreatic neuroendocrine neoplasms (GEP-NEN). Recently, it has been shown that plasma levels of CgA correlate with tumor load and predict survival of patients with NEN of the small bowel. It is assumed that this is as well valid for NEN of the colon and rectum, however, this is not supported by data. To evaluate this assumption, we analyzed 62 patients with NEN of the colon and rectum listed in the Marburg GEP-NEN registry for clinicopathological characteristics, expression and plasma levels of CgA. The present study demonstrates that immunohistochemical CgA and synaptophysin are good markers for histological diagnosis in patients with NEN of the colon and rectum. However, plasma CgA is a poor marker to follow-up these patients because only a minority exhibited increased levels which did not increase significantly during tumor progression. In contrast to NEN of the small bowel, there is no correlation of CgA plasma levels with tumor burden or survival. Patients with NEN of the colon and rectum displayed a relatively good prognosis resulting in a median survival of 8.5 years. However, a subset of patients affected by G3 neoplasms, exhibited a poorer prognosis with a median survival of 2.5 years. Taken together, CgA is a valuable marker for immunohistochemical diagnosis, but CgA plasma concentration is not suitable to mirror tumor burden or prognosis in patients with NEN of the colon and rectum.

## Introduction

Gastroenteropancreatic neuroendocrine neoplasms (GEP-NEN) represent a rare tumor entity, accounting for less than 1% of all malignancies. They occur in the gastrointestinal tract, arise mostly in the stomach, rectum, and small intestine and include functional and non-functional tumors [1–3]. Functional GEP-NEN differ in clinical symptoms due to the secreted hormones. But there is rising evidence that non-functional GEP-NEN also differ in their biological behavior, e.g. onset and pattern of metastasis or responsiveness to chemotherapy according to the localization of the primary tumor lesion [4]. Thus, a similar biological behavior occurs in tumors originating from neuroendocrine cells of similar embryological structures or organs e.g. small bowel or pancreas [5]. Due to the lack of confirmed information about NEN of the colon and rectum, it is assumed that this tumor entity may behave similarly to those tumors originating from the small intestine.

Usually, NEN are well-differentiated tumors exhibiting a slow growth associated with a good prognosis. However, some of these tumors grow more rapidly, resulting in less favorable survival rates. In fact, there are currently no well-established prognostic markers for patients with these tumors [6]. Histological staining for Ki-67, the degree of differentiation of the tumor, the presence of metastases, or lymph node involvement are currently the most reliable markers to predict tumor growth and survival of patients with neuroendocrine tumors [7–9].

In the past, several plasma, serum and urine markers have been evaluated as predictors of tumor progression in NEN, such as chromogranin A (CgA) [10, 11], serotonin [12], 5-hydroxyindoleacetic acid (5-HIAA) [13], neurokinin A [14], neuropeptide K [15], neuron-specific enolase [16], or E-cadherin [17]. Currently, CgA is widely used in the clinical routine for diagnosis and follow-up of patients with GEP-NEN. CgA belongs to a family of secretory proteins with ubiquitous distribution in many normal and neoplastic neuroendocrine cells. Several groups have elucidated a correlation between plasma levels of CgA and disease severity. Thereby, significantly higher levels of CgA have been reported in metastatic situations compared with nonmetastatic disease [18]. Moreover, our group has shown in 344 patients with GEP-NEN that increased plasma levels of CgA predict a shorter survival. In addition, a sudden increase of plasma CgA was associated with an unfavorable outcome [11]. There was also a positive correlation between plasma levels of CgA and tumor burden. As metastases in patients with GEP-NEN occur mostly in the liver, it is not surprising that enhanced levels of CgA correlate with hepatic tumor burden [10]. However, there is rising evidence that the tumor localization may determine the total amount of serum levels of CgA, as patients with Zollinger Ellison Syndrome (gastrinoma) show much higher levels of plasma CgA compared to patients with GEP-NEN of the small bowel [19].

Plasma CgA-levels have been suggested to indicate the presence of neuroendocrine tumors with high sensitivity and specificity (70% to 93%) [13]. However, several other tumors such as pheochromocytoma, prostate cancer, neuroblastoma, or small-cell lung cancer can also express CgA. Moreover, elevated plasma CgA levels may occur in a variety of conditions unrelated to the presence of a malignant tumor. A more specific tumor marker for neuroendocrine tumors represents serotonin or its metabolite 5-hydroxyindoleacetic acid (5-HIAA) determined in serum and/or 24h urinary samples. The specificity of urinary 5-HIAA can be up to 90% but the sensitivity in all neuroendocrine tumors is very low with only 35% [20, 21] reflecting that only subgroups of neuroendocrine tumors reveal increased amounts of serotonin or 5-HIAA. In patients with NEN of the small bowel, elevation of 5-HIAA shows a high sensitivity as nearly two thirds of these patients exhibit increased urine levels of 5-HIAA. Thereby, elevated serum levels of either 5-HIAA or serotonin are associated with an increased incidence of carcinoid syndrome and/or carcinoid heart disease [22, 23].

The aim of this study is to characterize clinicopathological hallmarks and biomarkers such as CgA in diagnosis and follow-up of patients with colorectal NEN. Furthermore, it should be investigated whether CgA is a suitable marker to predict survival or tumor progression in patients with NEN of the colon and rectum.

## Patients and methods

### Patients

In 1998, a prospective database for patients suffering from gastroentero-pancreatic neuroendocrine neoplasia (GEP-NEN) was established at the University Hospital of Marburg. Our prospective database of patients with GEP-NEN was screened for patients with NEN of the colon and rectum. The screening led to the identification of 62 individuals treated at the University Hospital of Marburg. The screening revealed information about clinical manifestations, histopathological findings, radiological findings, laboratory results, treatment, and survival.

None of these patients was diagnosed by chronic atrophic gastritis. Renal function test was performed in all of the patients enclosed in this study. Higher grades of renal insufficiency (grade 3 and 4) led to drop out of evaluation of plasma CgA levels. None of the patients had reported a chronic use of proton pump inhibitors (PPI). Sporadic use of PPI was no exclusion criterium. In case of increased plasma CgA levels PPI medication was discontinued and the CgA test was repeated or the patient was excluded if discontinuation was not feasible.

When CgA decreased or increased more than 30% a biochemical response or progression was postulated.

Collection, storage, and evaluation of patient related information in our prospective GEP-NEN database was done with patient informed consent and with the approval of the local ethics committee at the University of Marburg. Participants provided their written informed consent to have their data included in the database and to the use of their data for research purposes.

### Statistical evaluation

All data are presented as mean ± standard deviation. Two-tailed paired Student's t test was used for statistical evaluation of the data. The one-way and non-parametric ANOVA test was used to calculate the p value for more than two groups. Median patient survivals were evaluated by Kaplan-Meier analysis [24]. Differences were tested by log-rank test. All calculations were performed with GraphPad Prism software. A p value < 0.05 was considered as statistically significant. The comparisons between tumor characteristics and laboratory features were based on Fisher’s exact tests.

### Histological diagnosis

The diagnosis of NEN was made after histological staining of surgically resected or endoscopically removed tumor samples which had been routinely fixed in formalin and embedded in paraffin. Chromogranin A and synaptophysin were determined immunohistochemically on paraffin sections after heat-induced antigen retrieval. CgA was detected by a polyclonal rabbit antibody (code no. A 0430; Dako, Hamburg, Germany), synaptophysin by a monoclonal mouse antibody (clone SY38; code no. M 0776; Dako, Hamburg, Germany). Immunostaining was performed using a standard avidin biotin complex (ABC)-peroxidase method with 3,3’-diaminobenzidine (DAB) as chromogen. The immunhistochemical staining of the tumor samples for CgA and synaptophysin was qualitatively scored as follows: “negative” (no visible staining), “weakly or focally positive” (up to 30% positive tumor cells), “positive” (30 to 75% positive tumor cells), and “highly positive” (more than 75% strongly positive tumor cells) staining.

### Chromogranin A enzyme linked immunoassay

CgA from EDTA plasma samples was determined by using an ELISA kit of Dako (Glostrup, Denmark). In this assay, a rabbit polyclonal antibody directed against a 23 kDa C-terminal fragment of human chromogranin A was used. The chromogranin A standards of the kit were calibrated against the C-terminal fragment. Median and normal range as indicated by the manufacturer is 10 U/l (range 2–18 U/l). A cut-off for significantly elevated plasma levels of chromogranin A has been defined at 50 U/l in a previously published work [11]. Chromogranin A levels were exclusively measured in the laboratories of the Centre of in vitro diagnostics—Endocrinology at the University Hospital of Marburg using an ELISA which has been used without modifications since January 1995.

### Serotonin and 5-HIAA enzyme linked immunoassay

Before determination of serotonin and 5-HIAA patients were asked to avoid foods that contain high levels of serotonin 3 days prior collection. The concentration of serotonin was measured in a platelet-free serum by using an ELISA from DRG Instruments according to the manufacturer instructions (DRG-Instruments Marburg, Germany). Serum concentrations above 450 ng/ml were considered as significantly elevated. In case of multiple determinations the highest concentration of serotonin is reported. The concentration of the serotonin degradation product 5-HIAA was determined by using HPLC analysis with an electrochemical detector from Chromsystems Instruments according to the manufacturer instructions (Chromsystems Instruments & Chemicals Munich, Germany). Urine elimination per day of 5-HIAA above 47.1 μmol/d was considered as significantly elevated. In case of multiple determinations the highest value measured for 5-HIAA urinary excretion is reported in each patient.

### CEA and CA19-9 enzyme linked immunoassay

The CEA and CA19-9 concentration in the serum was measured by using the electrochemiluminescence immunoassay from Roche according to the manufacturer’s instructions (Roche Diagnostics, Mannheim Germany). Serum levels above 5ng/ml and 27U/ml, respectively, were considered as significantly elevated. In case of multiple measurements the highest level is reported.

## Results

### Clinical and histopathological features of colorectal NEN

**Fig 1:**
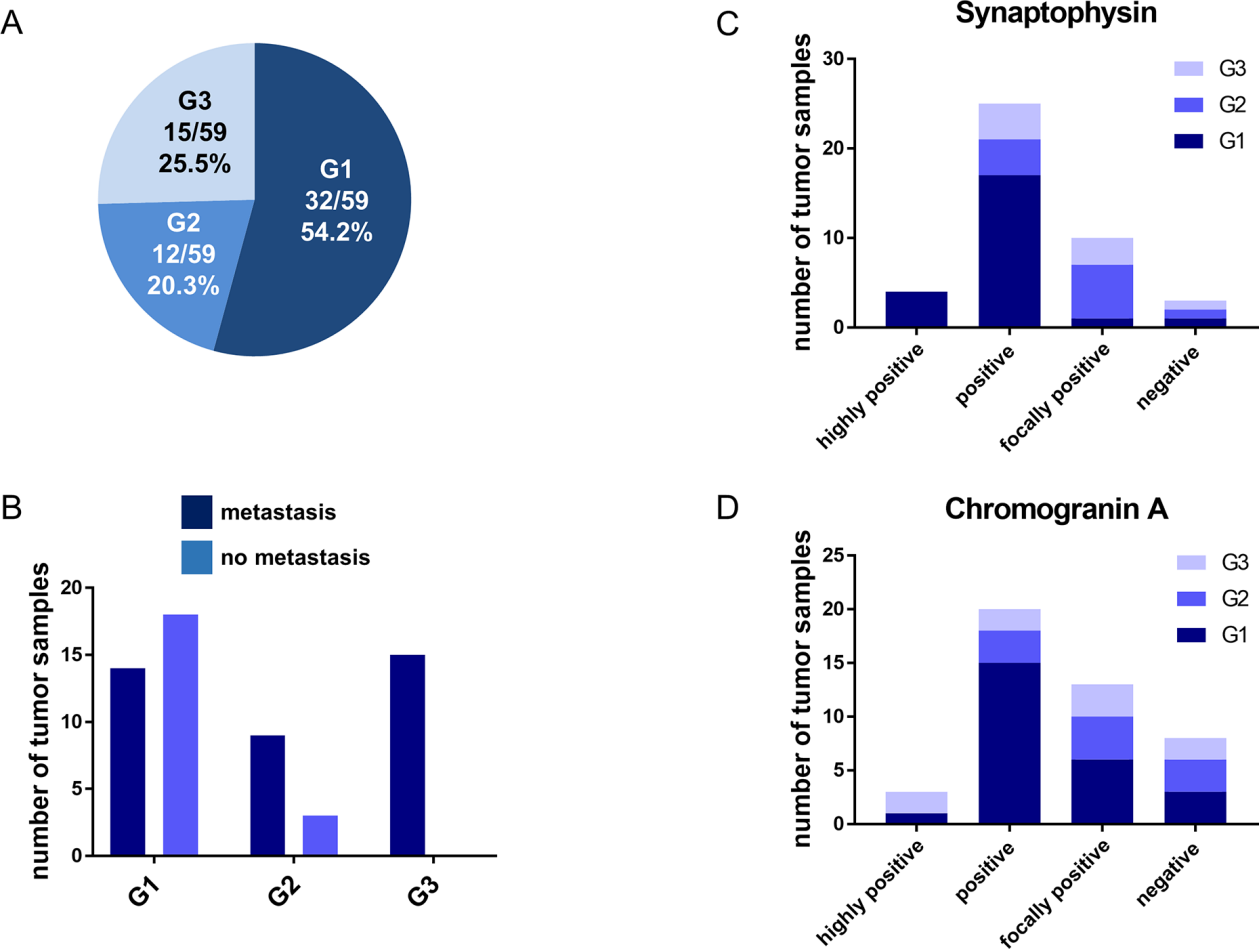
Histopathological features of colorectal GEP-NEN. A: Quantification of histological grading of NEN of the colon and rectum according to WHO criteria. B: Quantification of metastatic behavior of colorectal NEN with respect to histological grading. G1:14 M1 vs. 18 M0; G2: 9M1 vs. 3 M0 and G3 15 M1 vs. 0 M0 cases; M1: metastasis; M0: no metastasis. C and D: Quantification of immunohistochemical CgA (n = 44) and synaptophysin (n = 42) staining in patients with colorectal NEN. The expression levels include “negative” (no visible staining), “weakly or focally positive” (up to 30% positive tumor cells), “positive” (30 to 75% positive tumor cells), and “highly positive” (more than 75% strongly positive tumor cells) staining.

**Fig 2:**
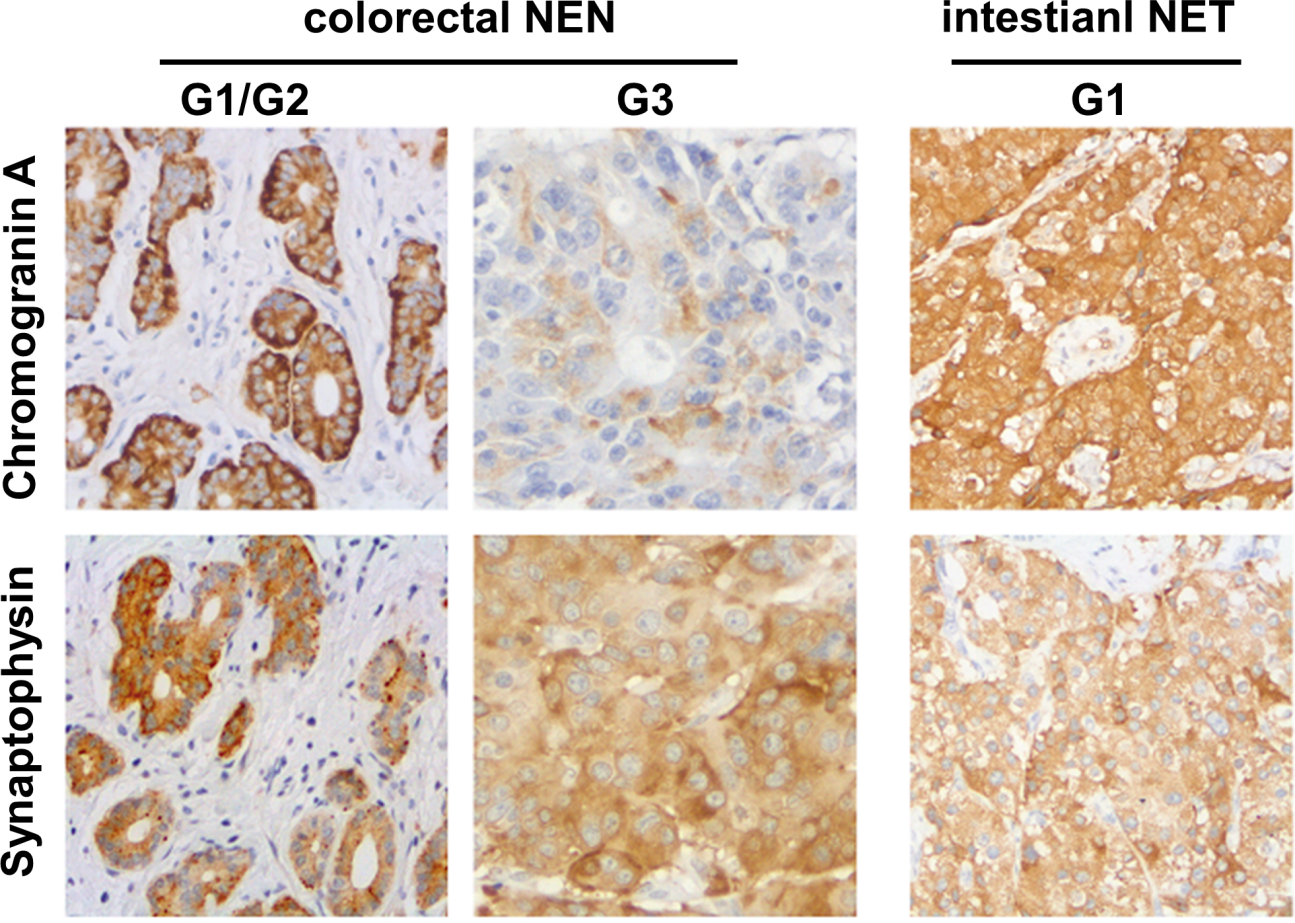
Immunohistochemistry in colorectal and intestinal NEN. Representative histological sections of well-differentiated (G1 and G2) neuroendocrine tumors and poorly differentiated colorectal NEN (A) and well-differentiated (G1) NET of the small bowel (B) are displayed after immunohistochemical staining for CgA and synaptophysin.

Analysis of our GEP-NEN database revealed 62 NEN patients with primary tumor localization in the colon or rectum. The clinical and histopathological details of these patients are depicted in Table 1. 61.3% of these patients (38/62) were male. Mean age at the time of diagnosis was 57.0 ± 15.0 years. The primary tumor localization was in the rectum in 41 (66.2%) and in the colon in 21 (33.8%) patients. Detailed histopathological information was available in 59 patients. The majority of tumors at this localization were well-differentiated (G1, 54.2%; G2, 20.3%), whereas poorly-differentiated neoplasms (G3, 25.5%) occurred with lower frequency (Fig 1A). G3 neoplasms were more frequently localized in the colon than in the rectum (52.4% vs. 9.5%; 11/21 vs. 4/41; P = 0.008). More than 64% (40/62) of the patients exhibited metastases at diagnosis, most frequently in lymph nodes and in the liver. Metastases were seen in less than half of the G1 tumors (43.5%) but were nearly always detectable in G2 (75%) or G3 (100%) tumors (Fig 1B). There was a significant correlation of G2 plus G3 neoplasms with metastatic disease in comparison to G1 tumors (P = 0.0004). To confirm the histological diagnosis of GEP-NEN in the foregut and midgut, several marker proteins, such as synaptophysin or CgA play a crucial role in the clinical practice. Proof of either expression of synaptophysin or CgA is required to confirm the diagnosis of a GEP-NEN. In our case series, all cases of GEP-NEN of the colon and rectum exhibited expression of at least one or both marker proteins. Eight (18.1%) of 44 and 3 (7.1%) of 42 specimens were negative for CgA and synaptophysin, respectively (Fig 1C and 1D). Well-differentiated tumors (G1 and G2) showed strong staining for the neuroendocrine markers in the tumor cell compartment, similar to GEP-NEN of the small bowel as presented in representative examples (Fig 2). Out of 35 G1 and G2 tumors 19 (54.3%) and 25 (71.4%) were highly positive or positive for CgA and synaptophysin, respectively. In less differentiated neoplasms (G3) of the colon and rectum the staining for either synaptophysin or CgA was mostly focal or negative (50% and 55.6%) but less intense compared to the well-differentiated tumors (Fig 1C and 1D). However, this trend failed to reveal statistical significance.

**Table 1:** Clinical characteristics of 62 evaluated patients with NEN of the colon and rectum.

**Number of patients**	62
**Gender** (female/male)	24/38
**Age at diagnosis** (years)	57.0 (27–82)
<60	33 (53.2%)
≥60	29 (46.8%)
**Grading**	
G1	32 (54.2%)
G2	12 (20.3%)
G3	15 (25.5%)
**Ki-67**	
mean (range)	21% (1–90%)
median	4%
**Metastases**	
number of patients with metastases	40 (63.5%)
liver	32 (50.8%)
bones	31 (49.2%)
lymph nodes	11 (17.5%)
lung	6 (9.5%)
other	12 (19.0%)
**SMS-receptor status**	
positive	26/45 (57.8%)
negative	19/45 (42.2%)
not performed	17
**Tumor resection**	
surgical resection	38/62 (61.3%)
endoscopic resection	14/62 (22.6%)
no tumor resection	6/62 (9.7%)
unknown	4/62 (6.4%)

### Impact of biomarkers during disease progression

**Fig 3:**
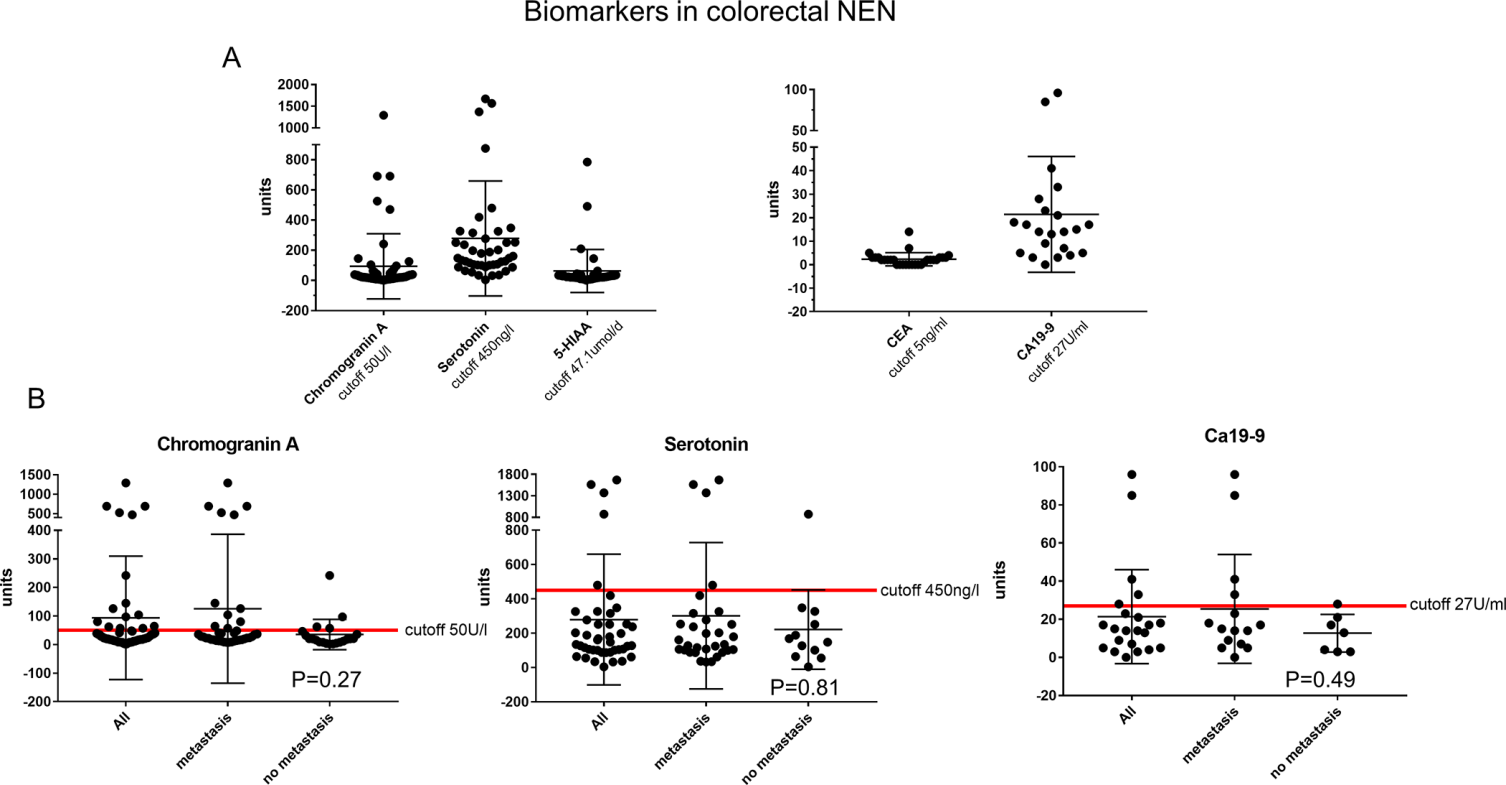
Maximum levels of tumor markers tested in patients with colorectal NEN. Maximum plasma levels ever measured at our hospital for the individual GEP-NEN patients for CgA (n = 59), serotonin (n = 42), urine elimination of 5-HIAA (n = 39), CEA (n = 29) and CA19-9 (n = 21) are displayed (A). Representative evaluations of CgA, Serotonin and CA19-9 in consideration of metastatic disease are shown in Fig 3B. The red lines represent the cutoff levels for significant elevation of the individual tumor markers (CgA (50 U/l), serotonin (450 ng/ml) and CA19-9 (27U/ml)).

As shown in Fig 3A, only 15 out of 59 patients (24.4%) displayed elevated plasma CgA levels above the significant threshold of 50 U/l. Rare events with significantly increased plasma levels of CgA were found at both tumor localizations (colon: n = 5; rectum: n = 10) and did not depend on tumor grading (G1: 8, G2: 3, and G3: 4). Furthermore, no correlation between elevated plasma CgA levels and immunohistochemical positivity in tissue sections was verified. Additionally, other tumor markers frequently increased in NEN of the small bowel were not significantly elevated in patients with NEN of the colon and rectum. In fact, we found only 5 cases with increased levels for 5-HIAA and serotonin in each group respectively (Fig 3A). Besides neuroendocrine markers in some cases CEA (n = 29) and CA19-9 (n = 21) were measured initially. Since CEA is most frequently increased in patients with colorectal cancer, in our group only 3 patients (10.3%) showed an elevated serum level (Fig 3A). Similar results were obtained for CA19-9, where only 22.7% of patients presented high levels (Fig 3A). There was no correlation between marker elevation and clinicopathogical information, in particular, regarding metastatic versus local disease (Fig 3B).

**Fig 4:**
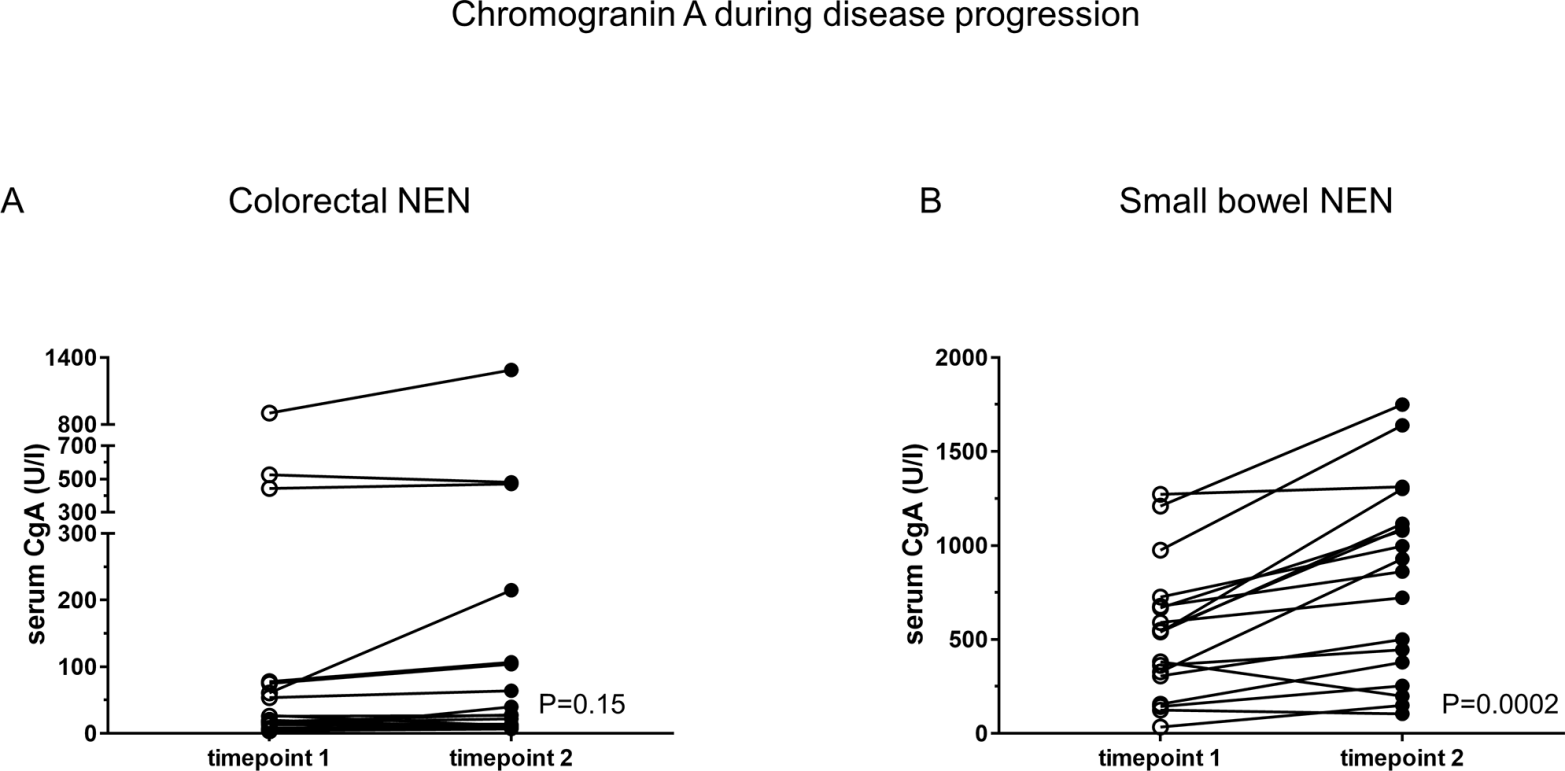
Plasma CgA levels in patients with confirmed tumor progression. Matched pairs of plasma CgA are shown before (timepoint 1) and after (timepoint 2) a confirmed tumor progression in patients with colorectal NEN (A, n = 20) and with NEN of the small bowel (B, n = 18). CT or MRI scans confirmed tumor progression according to RECIST criteria. The interval of tumor progression ranges from six to twelve months.

As the single determination of a tumor marker represents only a momentary view on the tumor, and levels of a tumor marker below a cut-off are possibly due to low tumor load, we evaluated plasma levels of CgA during follow up of patients with metastatic colorectal NEN. We compared the baseline CgA levels (Fig 4A, timepoint 1) with the CgA plasma levels of the same patients after tumor progression had occurred (Fig 4A, timepoint 2) which was verified by MRI or CT-scan according to RECIST criteria. Tumor progression occurred within 6 to 12 months. However, the increase in CgA levels was very low, with a mean of 31.2 U/l and most samples still remained under the cut-off value of 50 U/l (65%, 13/20 patients) (Fig 4A). To confirm this negative result with positive controls, we chose a control group of patients with NEN of the small bowel who had been treated within two incidentally selected consecutive months in our hospital and who also showed a tumor progression (confirmed by MRI or CT-scan) within 6 to 12 months. As demonstrated in Fig 4B, 12 out of 18 (66.7%) patient samples showed a highly significant increase in plasma CgA levels (P = 0.0002) during tumor progression which was in total nearly nine times higher (mean increase of 291.3 U/l) compared to samples derived from patients with NEN of the colon and rectum. Moreover, when assessing biochemical progression, there was a significant difference between both groups. Only 2 of 20 patients (10%) in the colorectal group, but 12 of 18 (66.7%) in the small bowel cohort presented biochemical progress (P = 0.0005) validating the limited impact of CgA in colorectal NEN patients.

### Outcome of colorectal NEN patients

**Fig 5:**
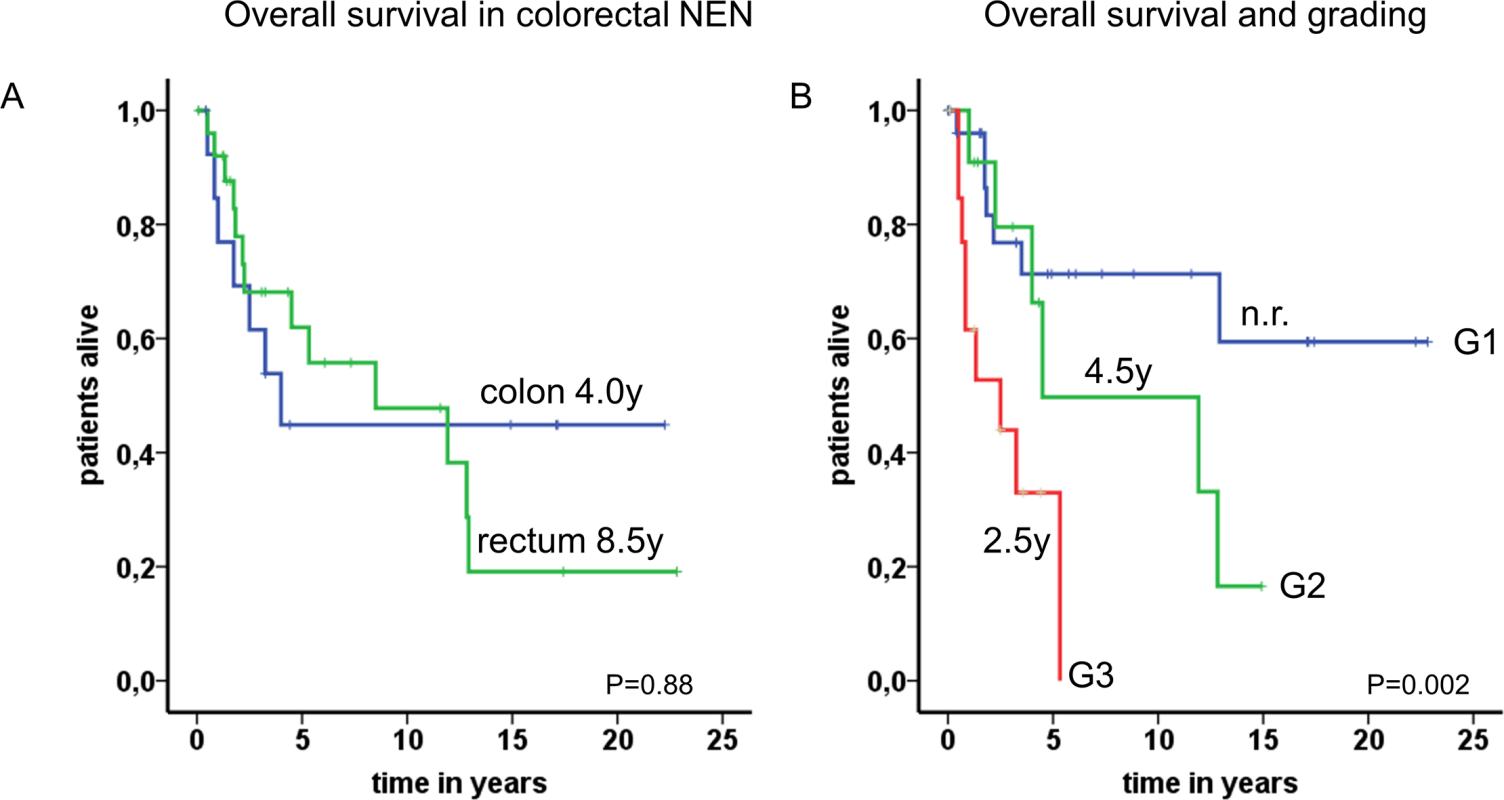
Survival rates of patients with colorectal NEN. A: A Kaplan-Meier plot displays the overall survival of 56 patients with colorectal NEN (median OS colon 4.0 years and 8.5 years for rectum). B: A Kaplan-Meier plot shows the survival analysis of three different subgroups based on grading who differ in their prognosis: The first group (30 patients, blue line, G1 tumor, Ki67: <2%, median OS not reached (n.r.)), the second group (11 patients, green line, G2 tumor, Ki67: ≤20%, median OS 4.5 years) displayed an intermediate prognosis and the third group (14 patients, red line, G3 tumor, Ki67: >20%) exhibits the worst prognosis with a median OS of 2.5 years.

The overall survival of all evaluated patients with NEN of the colon and rectum was relatively good, with a median overall survival of 8.5 years. In our series patients with GEP-NEN originating from the rectum generally had no better prognosis compared to patients with a primary localization of the NEN in the colon (mOS 8.5 years v.s. mOS 4.0 years; P = 0.88)(Fig 5A). There was a trend towards separating curves during the first 5 years, which however did not reach significance due to multiple patients lost to follow-up in the colon group. However, we have identified three subgroups differing markedly in prognosis based on grading. As shown in Fig 5B, patients with G1 tumors (blue line) presented the best outcome. Even after 10 years the survival rate was nearly 75%.

The second subgroup (Fig 5B, green line) of G2 graded tumors showed an intermediate prognosis with a mOS of 4.5 years and patients with G3 neoplasms revealed a poor prognosis with a mOS of 2.5 years. The latter group is characterized by a high proliferation rate above 20% and consisted of poorly differentiated neoplasms in the majority of cases. With respect to statistical evaluation, the outcome was significantly influenced by grading (P = 0.002). Furthermore, metastatic disease was borderline significant as negative predictor for survival (metastatic vs. non-metastatic; 5.3 years vs. not reached; P = 0.05). Additional parameters such as age, SMS-receptor status, tumor size, positive lymph nodes and biomarkers failed to reveal a significant correlation to outcome in univariate analysis.

## Discussion

The results of this study demonstrate that CgA is a valuable marker for histological diagnosis of NEN of the colon and rectum, but a very poor plasma marker for follow-up patients suffering from this type of cancer. This is in contrast to results obtained from patients with NEN of the small bowel, as recently published by several groups [11, 14, 25]. Patients with NEN of the small bowel exhibit increased plasma levels of CgA and show a strong correlation of plasma CgA with tumor load and overall survival. This qualifies CgA as a prognostic parameter in these patients. Also for patients exhibiting a primary tumor localization in the pancreas or duodenum, a correlation of plasma CgA and tumor load has been reported [26, 27]. However, there are several other components influencing the plasma levels of CgA in most of these patients. Atrophic gastritis, which often results in increased gastrin levels, is characterized by elevated CgA plasma levels. Similarly, the application of proton pump inhibitors, which is the symptomatic treatment of choice in patients with gastrinoma, increases plasma levels of CgA. These influencing factors impair the value of CgA as a prognostic parameter in patients with NEN of the stomach and duodenum.

As reported in our study and in contrast to tumor located in the small bowel and pancreas, plasma levels of CgA in colorectal NEN are not significantly elevated. Even after confirmed tumor progression with increasing tumor load in the liver, in most patients plasma levels of CgA were not or only slightly elevated but do not exceed the threshold of 50 U/l. As expected, there is no correlation of plasma CgA level and overall survival or tumor progression in patients with NEN of the colon and rectum.

The lack of variation in CgA plasma levels in patients with colorectal NEN are not due to technical issues concerning the assay or the examined material, since CgA plasma levels of patients with NEN of the small bowel measured with the same system were strongly elevated. Currently, there are four commercial assays available showing altogether a linear correlation between serum and plasma CgA levels with variations in the absolute levels in individual tumor entities [28, 29]. The sensitivity of the kits in measuring elevated CgA serum levels varies between 67% to 93% and the specificity between 85 to 96% [28]. In principle, detection of CgA serum levels can diagnose NEN and predict tumor growth or prognosis in patients with some types of NEN [30]. However, the lack of international standards for CgA plasma levels and comparable antibodies impairs the comparison of CgA levels based on different kits [31].

The reason for the different behavior of circulating CgA in patients with NEN of the small bowel and the colon and rectum remains unclear. As recently suggested, the distribution of certain types of endocrine cells–EC cells and L cells—in the tubular gut, which are most probably the progenitors of the cancer cells, could explain this discrepancy [32]. EC cells, expressing large amounts of CgA, are found predominantly in the small bowel and decrease in frequency in the colon. L cells express only small amounts of CgA, occur mostly in the large intestinum and increase along the colon [33, 34]. The small amounts of CgA expressed in L cells are probably sufficient to reveal a positive signal in immunohistochemistry but not enough to lead to a significant rise of CgA plasma levels documenting a significant tumor growth. Whereas in intestinal NETs we usually find a homogeneous and strong staining for CgA, in our colorectal cohort a relevant proportion of the patients (13/44 = 30%) only displayed focal positivity and 8 cases were negative (Fig 1). A specificity problem of the commercial CgA antibodies concerning detection of the related family members CgB or CgC cannot be excluded either. If we assume that EC or L cells are progenitors of the neuroendocrine tumor cells, the hypothesis that the number of CgA producing EC cells decreases along the colon could explain why only a small proportion of patients in our collection of colorectal NEN exhibit significantly elevated CgA plasma levels, while the majority of our patients with colorectal NEN does not. Another reason could be the lack of a strict relation between expression and secretion. This phenomenon is well known for e.g. glucagon. Whereas a significant proportion of pNETs displays positivity for glucagon in immunohistochemical staining a significant secretion leading to a glucagonoma syndrome is a rare event [35]. Therefore, low expression levels of the respective marker may be detectable by immunohistochemistry in tissue sections, though will not necessarily lead to detectable, elevated plasma levels of the marker.

A higher tumor load in patients with small bowel NEN as a reason for significantly higher plasma CgA levels can by excluded, because most patients with colorectal NEN showed an advanced, metastatic tumor stage. In contrast to epidemiological data [1, 36], in our study most patients with rectum NEN showed an advanced or metastatic disease. We suppose this phenomenon is related to the referral of patients with more advanced disease to our ENETs center and also might bias the non-significance between overall survival of colon and rectum NEN. In the SEER database more than 90% of patients presented with localized disease, whereas lymph node and distant metastasis were rarely present [1]. However, grading-based evaluation revealed that most patients with G1 tumors are alive more than ten years after diagnosis and their survival rate possibly may not differ significantly from the general population, which is in line with previous publications [1, 3].

In the present study we can also show that other widely used tumor markers in patients with NEN of the tubular intestine, serotonin or its degradation product 5-HIAA, are ineffective to document tumor growth or predict survival in patients with NEN of the colon and rectum. This is in line with findings of other groups who reported a correlation of serum levels of 5-HIAA and tumor load and/or survival only in patients with small bowel NEN but not in patients with colorectal NEN [22, 23]. As elevated levels of 5-HIAA are strongly correlated with the risk of developing carcinoid heart disease (CHD), it is not surprising that patients with metastatic colorectal NEN rarely exhibit signs of a CHD. This is much lower compared to patients with NEN of the small bowel who reveal high amounts of 5-HIAA and suffer from CHD in 25–60%, which is associated with a shortened survival [14, 22, 23]. As colorectal malignancies can secret other biomarkers such as prostate specific acid phosphatase, PYY, NPY, GLPs or somatostatin, our study was limited by its retrospective nature and reevaluation of additional serum markers were not feasible. However, besides the assessment of monoanalytes, previously a blood-based multianalyte neuroendocrine gene transcript assay (NETest) was introduced [37]. NETest was superior in predicting progressive disease and disease alterations compared to CgA [38, 39]. However, patients with colorectal NEN were not included in this pilot project and actually the NETest is not routinely available.

Since CgA plasma levels are not sufficient to predict tumor growth or survival in patients with colorectal NEN, we looked for other parameters to stratify these patients. The median overall survival (mOS) of our patients with colorectal NEN was 8.5 years with a 5-year survival rate of 64%. This is in-between published results reporting 62% for patients with rectal NEN or 75% for patients with NEN of the colon [40–42]. A detailed analysis of survival rates of patients with colorectal NEN led to three distinct groups that markedly differed in their prognosis and confirmed the value of the classification suggested by the WHO [43, 44]. The first group primarily comprises patients presenting with well-differentiated, small tumors mostly accidentally seen in colonoscopy. These well-differentiated tumors are predominantly located in the rectum and show no metastasis at diagnosis. A complete endoscopic excision of these tumors if smaller than 1 cm is a curative treatment, as shown in this study and recently by others [45, 46]. These patients do not require a standardized follow-up program because their individual risk to develop a relapse or other carcinoid tumors is not elevated [47]. A second group of patients, also showing well differentiated tumors with a low proliferation rate, differs from the first group mostly in the existence of metastasis at diagnosis and a slightly increased proliferation rate. Patients with G2 well-differentiated metastatic colorectal NEN exhibited a median OS of 4.5 years and are thus not comparable with patients with well-differentiated, metastatic small bowel NET [48]. However, in both tumor entities the predominant tumor load is in the liver resulting in comparable local treatment options [49].

A third group, comprising poorly differentiated, fast-growing neoplasms (G3) and widespread metastasis, is associated with poor prognosis with no patient alive after five years. This is comparable to poorly differentiated NEC derived from foregut or midgut exhibiting a median survival between 1.8 and 2.4 years [50, 51]. The location of the metastasis is scattered over the entire body, which is in contrast to predominantly hepatic metastasis pattern in patients with well-differentiated NEN of the intestine [51].

Taken together, our results clearly demonstrate that CgA is an appropriate histological marker to establish the diagnosis of neuroendocrine tumors in the colon and rectum. In contrast to other neuroendocrine tumor localizations, CgA plasma levels are not suitable to diagnose or follow up the wide majority of patients with colorectal NEN since CgA is rarely elevated and does not mirror tumor burden or predict survival in these patients. Whether the use of a recently suggested array-based, multifactor analysis of blood mRNA levels can overcome this problem in daily patient care must be evaluated in future studies [52].

## Supporting information

S1 Table Hindgut-NEN-data set. The basic database underlying this analysis is available as S1.
(XLS)
